# HBV reactivation in patients with rheumatoid arthritis treated with anti-interleukin-6: a systematic review and meta-analysis

**DOI:** 10.1093/rheumatology/kead243

**Published:** 2023-10-23

**Authors:** Stamatia Katelani, George E Fragoulis, Athanasios-Dimitrios Bakasis, Abraham Pouliakis, Elena Nikiphorou, Fabiola Atzeni, Theodoros Androutsakos

**Affiliations:** Department of Pathophysiology, Medical School, National and Kapodistrian University of Athens, Athens, Greece; First Department of Internal Medicine, Propedeutic Clinic, “Laiko” Hospital, National and Kapodistrian University of Athens, Athens, Greece; Institute of Infection, Immunity and Inflammation, University of Glasgow, Scotland, UK; Department of Pathophysiology, Medical School, National and Kapodistrian University of Athens, Athens, Greece; 2nd Department of Pathology, National and Kapodistrian University of Athens, Medical School, University General Hospital Attikon, Athens, Greece; Centre for Rheumatic Diseases, King’s College London, London, UK; Rheumatology Unit, Department of Experimental and Internal Medicine, University of Messina, Messina, Italy; Department of Pathophysiology, Medical School, National and Kapodistrian University of Athens, Athens, Greece

**Keywords:** tocilizumab, HBV, HBV reactivation, anti-IL-6, RA

## Abstract

**Objective:**

The objective of this study was to assess the possibility of HBV reactivation (HBVr) in patients with RA under anti-IL-6 treatment.

**Methods:**

Using PubMed, Scopus and EMBASE, we performed a systematic literature search for articles related to HBVr in RA patients under anti-IL-6 treatment. The search was performed with no date limits and was last updated 28 January 2023. The results from all the databases were combined and duplicates were excluded, as were non-English articles, case reports, position articles, comments, and paediatric studies.

**Results:**

Our initial search led to 427 articles; 28 were duplicates, 46 non-English, 169 reviews, 31 books/letters, 25 case reports, and 88 irrelevant to the meta-analysis aim; 21 were excluded due to inadequate information, leaving 19 articles, with a sum of 372 RA patients with chronic HBV (CHB) or resolved HBV infection, for further analysis. The overall risk for HBVr in RA patients with CHB was 6.7%, increasing to 37% when only RA patients with CHB and no antiviral prophylaxis were included. On the contrary, HBVr was close to 0% in RA patients with resolved HBV infection, irrespective of antiviral prophylaxis. All RA patients experiencing HBVr in these studies were successfully managed with antiviral treatment and/or drug withdrawal.

**Conclusion:**

Overall, anti-IL-6 treatment comes with a significant risk of HBVr in RA patients with CHB; risk is diminished when antiviral prophylaxis is used. In contrast, in RA patients with resolved HBV infection, the risk of HBVr seems to be extremely low. Large, well-designed studies (either controlled trials or multicentre/international observational studies) are warranted to further validate these results.

Rheumatology key messages

- HBV reactivation risk in RA patients with chronic hepatitis B was 37%.
- HBV reactivation risk in RA patients was almost 0% in patients with resolved HBV infection.
- No RA patients under antiviral prophylaxis suffered HBV reactivation.

## Introduction

Hepatitis B reactivation (HBVr) is a well-recognized problem in everyday practice in patients receiving immunosuppressive/immunomodulatory treatment, like those living with autoimmune inflammatory rheumatic diseases (AIIRDs) [1].

The definition of HBVr differs among various guidelines; however, it can be summarized as a HBV DNA rise of >10-fold to 100-fold in patients with detectable HBV DNA at baseline, or as HBV DNA and/or HBsAg appearance in patients who are HBV DNA– or HBsAg-negative at baseline, respectively [2, 3]. The clinical course of HBVr varies from a simple HBV DNA rise and HBsAg seropositivity without hepatitis, to mild reactivation with clinical hepatitis, or even fulminant liver failure with transaminasaemia, encephalopathy and coagulopathy, depending on pre-treatment HBV status, liver fibrosis, and patient comorbidities [2–4].

Various factors are associated with HBVr, including those related to HBV, to the host, and to any immunosuppressive/immunomodulatory treatment, and HBVr risk is broadly classified as high (>10%), moderate (1–10%), or low (<1%) [5]. This risk seems to be higher among patients with chronic HBV (CHB) (defined as HBsAg and/or detectable HBV DNA) than in those with resolved HBV infection, defined as HBV core antibody (HBcAb) seropositivity with negative serum HBsAg and HBV DNA [5–9].

For patients living with an AIIRD, HBVr risk seems higher in patients receiving B cell–depleting therapies like rituximab, even though this risk is probably lower when compared with that of patients with lymphoma [1, 3, 10–12]. The use of TNF inhibitors also poses a considerable risk, as does the prolonged use of CSs, especially in high doses [1, 5, 13–15], while HBVr risk seems to be low in patients under conventional DMARDs [1, 5]. Data regarding HBVr in patients under treatment with anti-IL-6 (one of the major bDMARDs used in RA) are scarce [16, 17]. Notably, IL-6 is a cytokine that controls a number of pathways, promoting also liver regeneration and hence playing a crucial role potentially in protecting against HBV infection [18, 19].

The aim of this systematic literature review and meta-analysis was to analyse the risk of HBVr in patients with RA under anti-IL-6 treatment.

## Materials and methods

### Method

The meta-analysis was conducted following the recommended items of Systematic Reviews and Meta-Analysis (PRISMA) guidelines [20] and Meta-analysis of Observational Studies in Epidemiology (MOOSE) guidelines [21]. The Newcastle–Ottawa scale (score 0–9) was used to assess the quality of the non-randomized controlled trials (RCTs) [22]. The quality of the studies was assessed by two investigators (S.K., T.A.), and in the case of disagreement consensus was reached upon discussion with a third assessor (G.E.F.). This article is based on previously conducted studies; the authors did not undertake any new studies involving human participants or animals.

### Study identification

We performed a systematic literature search for relevant articles using PubMed, Scopus and EMBASE. The search was performed with no date limits and was last updated on 28 January 2023. The search strategy focused on the following search terms: ‘tocilizumab‘ (All Fields) OR ‘sarilumab‘ (All Fields) OR ‘immunosuppression therapy‘ (MeSH Terms) OR ‘immunosuppressive therapy‘(All Fields) AND ‘HBV‘ (All Fields) OR ‘hepatitis b‘(MeSH Terms) OR ‘hepatitis b‘(All Fields) AND ‘arthritis, rheumatoid‘(MeSH Terms) OR ‘RA‘ (All Fields) OR ‘autoimmune diseases‘ (MeSH Terms) OR ‘autoimmune diseases‘ (All Fields) OR ‘rheumatic diseases‘ (MeSH Terms) OR ‘rheumatic diseases‘(All Fields). Reference lists of relevant articles were also reviewed.

The search results were screened by two independent reviewers (S.K., T.A.) using the titles and abstracts, and all articles considered relevant were evaluated in full-text format. In the case of a disagreement, consensus was reached after discussion with a third reviewer (G.E.F.). The results from all databases were combined, and duplicates were excluded, as were non-English articles, case reports, position articles, comments, and studies with paediatric populations.

### Statistical analysis

A meta-analysis was performed using both the fixed and random effects methods. Since it was not feasible to collect detailed information for each individual patient, the analysis was performed on the aggregated data; such data were extracted from the reported results of the studies after determination of their significance. The meta-analysis was conducted using the R statistical computing language (edition 4.2.2) [23], within the Windows (Microsoft) environment and using the specialized package meta for the R language [24, 25]. In the studies in which the mean value and s.d. were not reported, the median and first and third quartiles were used to estimate the mean value and s.d., as proposed by Hozo *et al.* [26]. An improved method suggested by Bland was also used in cases where the maximum and minimum values were reported [27]. For such estimations of the mean value and s.d., the software Deep Meta Tool, Version 1 was used [28]. If only the minimum and maximum values were reported, then the range rule was applied to estimate the s.d., and the mean value was considered equal to the median. Finally, for studies based on case series with detailed information for each patient, the mean value and s.d. were calculated. In studies reporting on multiple groups treated with different agents, these groups were included separately in the meta-analysis. Assessment for risk of bias at the individual study level was not performed; however, risk of bias was evaluated cumulatively by the relevant funnel plots.

## Results

### Search results

**Figure 1.**
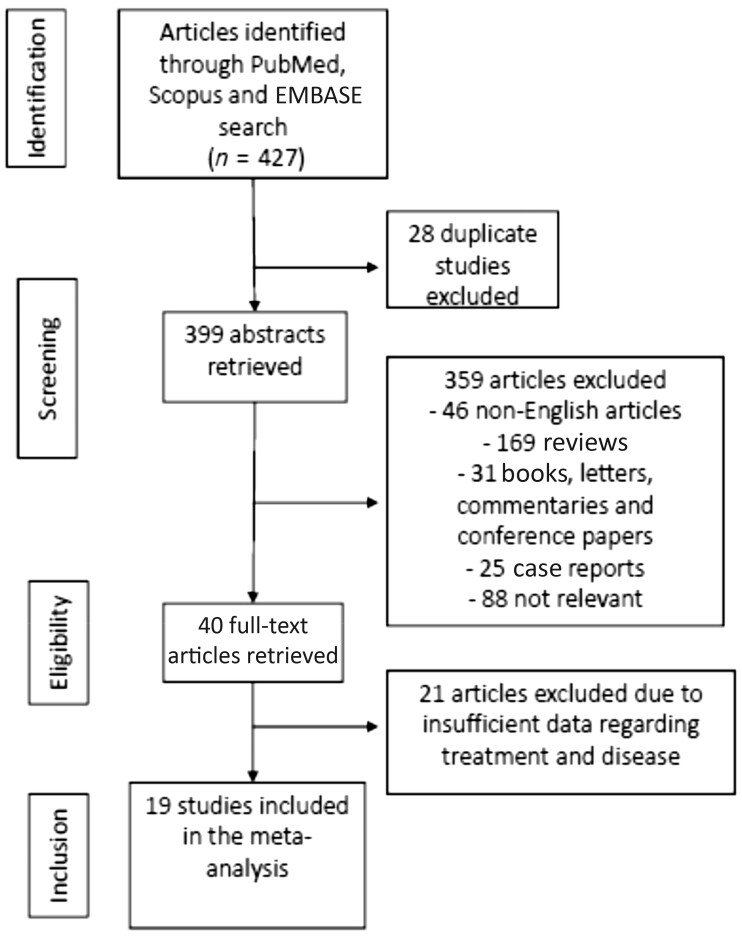
Flowchart for selection of articles. *n*: number

Our initial search led to 427 articles; 28 were duplicates, 46 non-English, 169 reviews, 31 books and letters, 25 case reports, and 88 were deemed to be irrelevant to the aim of the meta-analysis. From the remaining 40 articles, 21 were excluded due to lack of adequate information regarding treatment and disease, leaving 19 articles for further analysis (Table 1 [29–42] and Fig. 1). In the screening assessment, agreement between the researchers was 100%; regarding quality assessment, agreement between the researchers reached 98%; all studies were of medium to high quality.

**Table 1.** Studies included in the meta-analysis

Author, year	Design of study	Patients^a^ (total *n*)	HBV status before treatment	Prophylaxis (*n*)	HBV reactivation	Concomitant DMARDs/GCs treatment in HBVr patients
Mori *et al.*, 2011 [29]	Prospective	5	Resolved	No	No	–
Kato *et al.*, 2011 [30]	Prospective	1	Resolved	No	No	–
Urata *et al.*, 2011 [31]	Prospective	4	Resolved	No	1	MTX
Koike *et al.*, 2014 [32]	Prospective	52	Chronic or resolved	NS^b^	No	–
Barone *et al.*, 2015 [33]	Prospective	7	Resolved	No	No	–
Nakamura *et al.*, 2016 [34]	Retrospective	18	Resolved	No	2	Prednisolone (1), MTX (2)
Fukuda *et al.*, 2017 [35]	Prospective	48	Resolved	No	1	MTX
Chen *et al.*, 2017 [36]	Retrospective	2	Chronic	No	No	–
Chen *et al.*, 2017 [37]	Prospective	48	Chronic or resolved	2 with CHB (ADV, ETV)	3 with CHB	MTX (2), HCQ (3), SSZ (2)
Abdulaziz *et al.*, 2017 [38]	Retrospective	1	Resolved	No	No	–
Papalopoulos *et al.*, 2018 [39]	Retrospective	30	Resolved	2 (ETV, LMV)	No	–
Ahn *et al.*, 2018 [40]	Retrospective	15	Resolved	No	No	–
Schwaneck *et al.*, 2018 [41]	Retrospective	8	Resolved	No	1	MTX, prednisone
Watanabe *et al.*, 2019 [42]	Retrospective	25	Resolved	No	1	GCs, MTX
Lin *et al.*, 2019 [43]	Retrospective	11	Chronic	ETV or LMV	No	–
Serling-Boyd *et al.*, 2021 [44]	Retrospective	10	Resolved	NS	No	–
Kuo *et al.* 2021 [45]	Retrospective	71	Chronic or resolved	3 with CHB^c^	4 (3 CHB, 1 prior)	MTX (3), prednisolone (4), SSZ (3)
Hung *et al.*, 2021 [46]	Prospective	2	Resolved	NS	No	–
Chen *et al.*, 2023 [47]	Retrospective	14	Chronic	NS	1	NS

a Patients with RA treated with anti-IL-6.

b NS: Not specified.

c The antiviral drug hasn’t been specified. ADV: adefovir; ETV: entecavir; LMV: lamivudine; CHB: Chronic HBV infection; GCs: glucocorticoids.

### Meta-analysis

#### Patients included in the meta-analysis

A total of 372 patients with CHB or resolved HBV infection were included in the meta-analysis, with a median age of 64 years (range 18–91 years); data on gender were largely lacking; out of the 74 patients for whom gender was reported, 25 were male (33.8%). Forty-one patients had CHB and 279 had resolved HBV infection, with only 19 patients receiving antiviral prophylaxis upon anti-IL-6 initiation (Table 2). Fourteen patients experienced HBVr, with a median time between anti-IL-6 initiation and HBVr of 5.5 months (range 1–86 months). Of note, none of the patients experiencing HBVr were under antiviral treatment (Table 2). A total of 5 patients initiated antiviral treatment after HBVr, all with entecavir. No deaths after HBVr were reported.

**Table 2.** Characteristics of patients included in meta-analysis

Characteristics	*n* = 372
**Demogaphics**	
Male gender, *n*/*N*^a^ (%)	25/74 (33.8)
Age (years), median (range)	64 (18-91)
**HBV status**	
CHB, *n*/*N*^a^ (%)	41/320 (12.8)
Resolved HBV infection, *n*/*N*^a^ (%)	279/320 (87.2)
Antiviral prophylaxis, *n*/*N*^a^ (%)	19/294 (6.5)
Patients with HBVr, *n*/*N* (%)	14/372 (3.8)
**Concomitant treatment of patients with HBVr**	
MTX, *n*/*N*^b^ (%)	10/14 (71.4)
Prednisone-equivalent, *n*/*N*^b^ (%)	8/14 (57.1)
SSZ, *n*/*N*^b^ (%)	5/14 (35.7)
HCQ, *n*/*N*^b^ (%)	2/14 (14.3)
Antiviral prophylaxis, *n*/*N*^b^ (%)	0/14 (0)
**Time duration from anti-IL-6 treatment initiation and HBVr (months), median (range)**	5.5 (1–86)
**Post-HBVr antiviral treatment, n/N** ^b^ **(%)**	5/14 (35.7)
Entecavir, n/N^c^ (%)	5/5 (100)
**Outcome**	
Resolution, *n*/*N*^b^ (%)	14/14 (100%)

a Total number of patients for whom this information was available.

b Total number of reactivated HBV cases.

c Total number of patients who received antiviral treatment. CHB: chronic HBV; HBVr: HBV reactivation.

#### HBVr rates based on HBV status

In order to calculate HBVr risk based on a patient’s HBV status, we divided our patients into two groups: group 1 included patients with CHB and group 2 included patients with resolved HBV infection.

**Figure 2.**
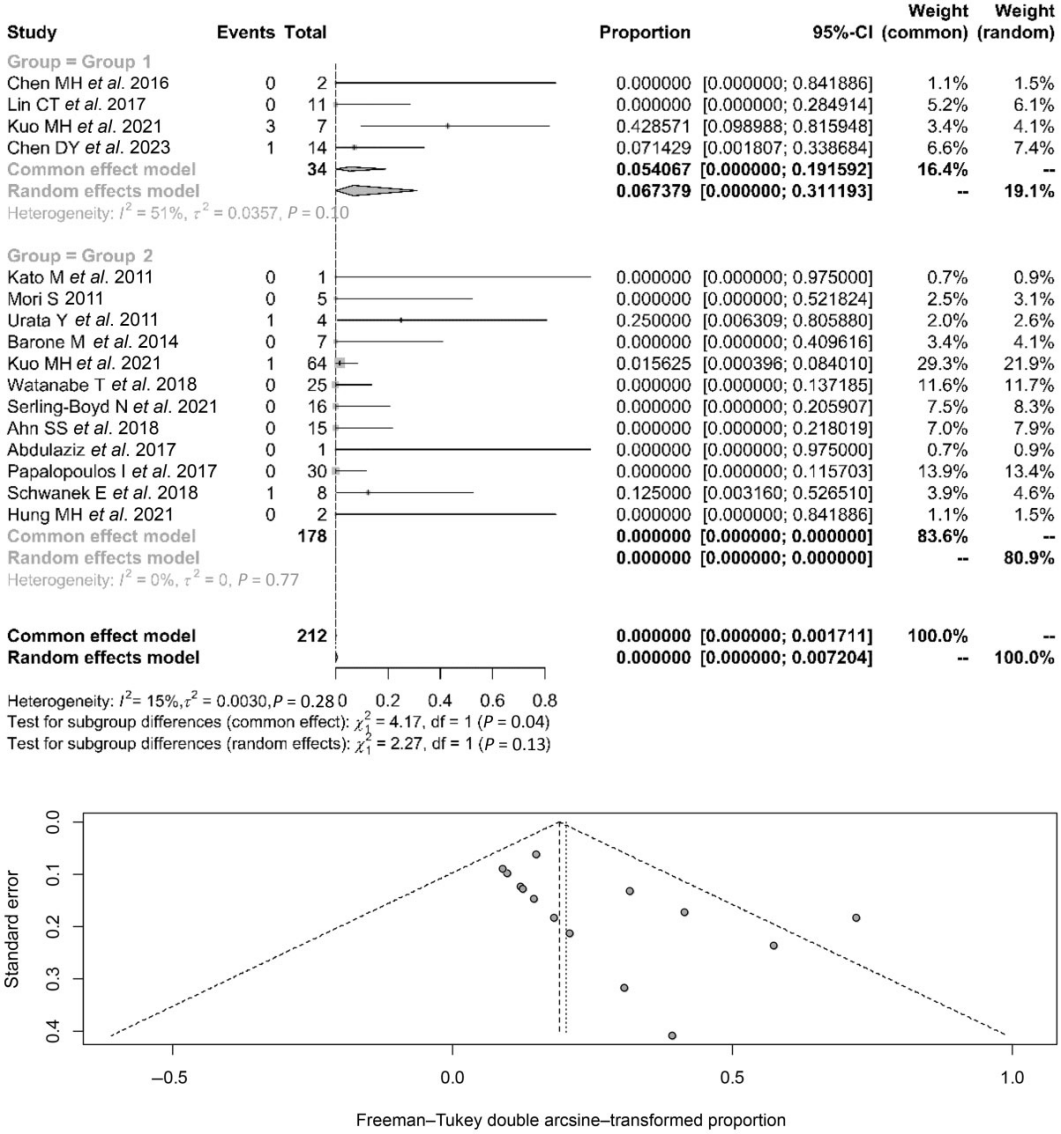
HBV reactivation rates in patients with CHB (group 1) and resolved HBV infection (group 2) and relevant funnel plot. The ‘Events’ column indicates the number of reactivation events; the ‘Total’ column indicates the total number of patients; the proportion corresponds to the relative proportion of reactivation events in the total population. CHB: chronic HBV

For both groups, the forest plot (Fig. 2) showed acceptable heterogeneity (*Ι*^2^ = 51%) for patients for CHB and excellent heterogeneity (*Ι*^2^ = 0) for those with resolved HBV; the publication bias was minimal for both groups. The aggregated percentage of HBVr for patients with CHB was 6.7% (95% CI: 0–31.1%) (5.4% for the fixed effects model), and almost 0% for patients with resolved HBV (of the 718 patients in this group, 3 experienced HBVr; however, according to both the random and common effects models, the overall reactivation proportion was 0% (95% CI: 0–0.9%). The comparison of the aggregated reactivation percentages indicated that there was a statistically significant difference, with patients with CHB showing higher reactivation rates than patients with resolved HBV infection (*P* <0.04, for the fixed effects models).

#### HBVr in patients without antiviral prophylaxis

**Figure 3.**
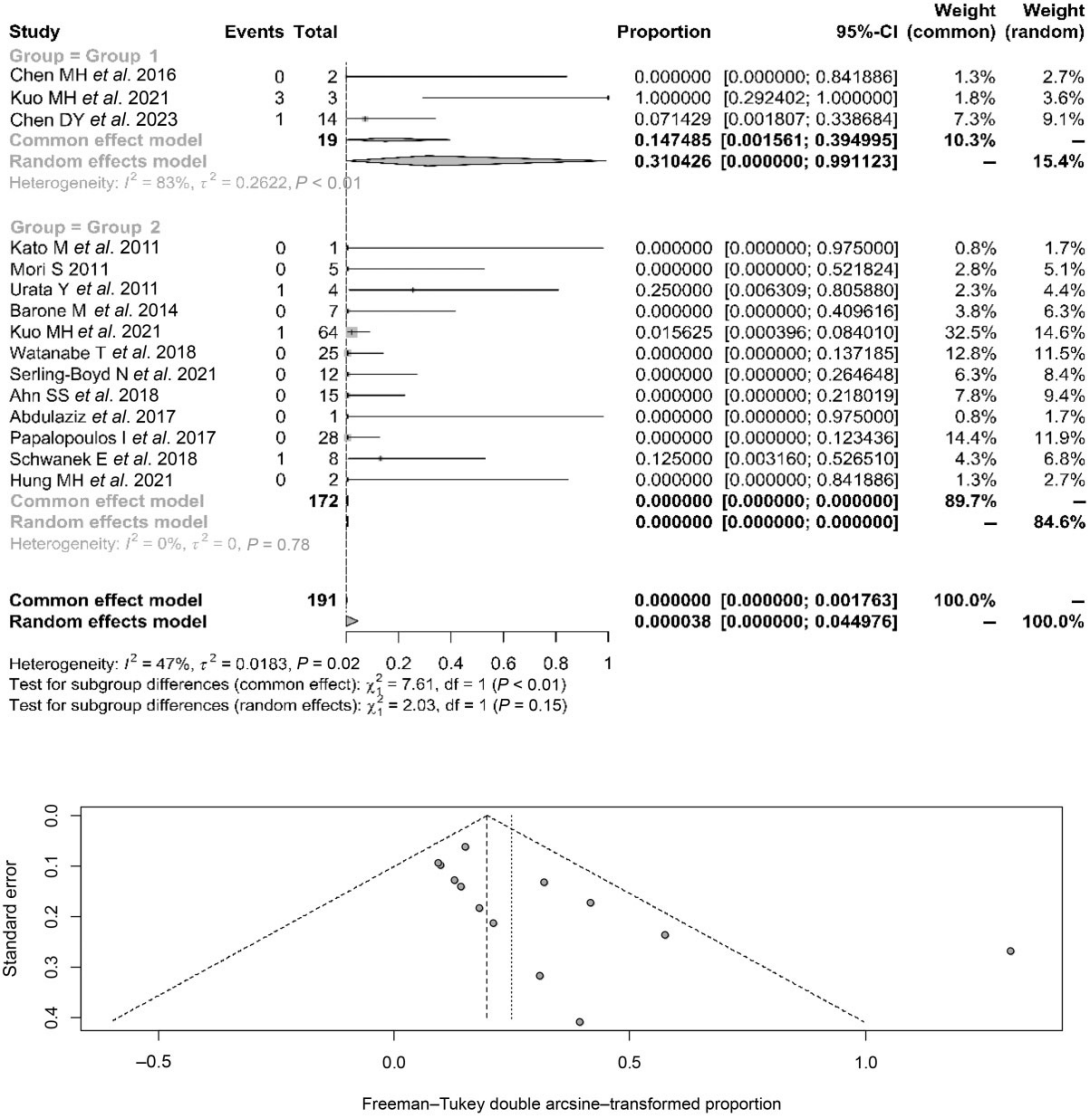
HBV reactivation rates in patients with CHB (group 1) and resolved HBV infection (group 2) with no antiviral prophylaxis and responding funnel plot. The ‘Events’ column indicates the number of reactivation events; the ‘Total’ column indicates the total number of patients; the proportion corresponds to the relative proportion of reactivation events in the total population. CHB: chronic hepatitis B

Fig. 3 depicts the findings for the patients who did not receive antiviral prophylaxis. There was poor heterogeneity among patients with CHB (83%) and excellent homogeneity among patients with resolved HBV infection (0%); the publication bias was minimal. The aggregated percentage of the HBVr for patients with CHB was 31% (95% CI: 0–99%) (14.75% for the fixed effects model), while for patients with resolved HBV infection, the aggregated percentage of recurrence was almost 0% (of the 172 patients in this group, 3 experienced recurrence, resulting in a percentage recurrence of 1.7%, 95% CI: 0–3.7%); however, according to both the common and random effects models, the overall reactivation proportion for the meta-analysis approach was 0%, with the 95% CI: 0%. The comparison of the aggregated reactivation percentages indicated that there was a statistically significant difference, with patients with CHB showing higher reactivation rates than patients with resolved HBV infection (*P* < 0.01, for the common effects model).

## Discussion

In this SLR and meta-analysis, we found that HBVr occurred in ∼7% of patients with CHB but was negligible in patients with resolved HBV infection. In the former group, HBVr risk rose up to 33% without anti-viral treatment administration. In contrast, HBVr remained trivial for RA individuals with resolved HBV infection who did not receive anti-viral treatment.

IL-6 is a pleiotropic cytokine exerting its action through a 130-kDa signal-transducing β-receptor (gp130), linked with either its membrane receptor (IL-6R) or its soluble receptor (sIL-6R) [48]. Apart from being a main mediator of inflammatory processes, IL-6 is also involved in homeostatic liver mechanisms. In fact, it promotes liver regeneration and protects liver cells from injuries caused by immune responses, alcohol, and viral infections [49, 50]. Moreover, IL-6 seems to play a crucial role in protecting against HBV infection. First, it seems to exert a dose-dependent inhibition of HBV entry into hepatocytes by downregulating sodium taurocholate co-transporting polypeptide (NTCP), most likely by inhibiting hepatocyte nuclear factor-4-alpha (HNF4a) expression [51, 52]. Second, it also seems to inhibit covalently closed circular DNA (cccDNA) formation in HBV-infected cells and to control HBx expression and HBV replication [19, 52]. Finally, IL-6 may also prevent cccDNA accumulation, making its role crucial for host defence against HBV infection [50]. Given the beneficial role of IL-6 in controlling HBV infection, the use of anti-IL-6 drugs could come with a certain risk of HBVr; however, data regarding this issue are scarce, limited mainly to case reports and small studies.

As mentioned earlier, the HBV status (chronic *vs* resolved) of the patient is one of the most critical factors affecting the risk for HBVr. Thus, recent EULAR recommendations for the screening and prophylaxis of AIIRD patients for chronic and opportunistic infections suggest that all patients who are starting treatment with immunosuppressants/immunomodulators should be screened for HBV status, examining in the first instance HBsAg, anti-HBcore and anti-HBs [53]. The same is also suggested by the recent EULAR recommendations for the use of IL-6 blockers in inflammatory conditions [54]. It has been established that the risk for HBVr in individuals who have chronic HBV and are starting treatment with bDMARDs is considerably high, and referral to a hepatologist for administration of anti-viral treatment is imperative [53]. A recent meta-analysis has shown that this also holds true for patients with inflammatory arthritis treated with TNF inhibitors [55]. However, data for other bDMARDs are limited. In this SLR and meta-analysis, we demonstrate that this is the case for RA patients treated with tocilizumab as well.

Regarding patients with resolved HBV infection, risk for HBVr appears to be much lower, and monitoring with liver function tests and HBV-DNA has been suggested over universal prophylaxis, in the recent EULAR recommendations [53]. However, meta-analyses or large studies in these patients receiving anti-IL-6 drugs are lacking. Our data offer additional evidence that prophylaxis does not seem to be necessary for these patients, since HBVr seems to be a rather rare event, with a rate close to 0%. Even though these drugs are relatively safe and given once daily, anti-viral prophylaxis in these patients is most probably not cost-effective; instead, close monitoring with HBV-DNA and/or liver function tests would be a reasonable approach [56]. Although the data are still limited, this seems to be the case for other biologic/targeted synthetic-DMARDs used for inflammatory arthritis, with the exception of rituximab, for which the risk for reactivation appears to be considerably higher [12, 55, 57, 58].

Our study has important strengths, as well as limitations. To the best of our knowledge, we present the first meta-analysis of HBVr in patients with RA under anti-IL-6 treatment. On the other hand, the number of studies included in our meta-analysis is not high. However, the heterogeneity of these studies is found to be high enough in our analysis, and the results seem to be adequate, in our opinion, to draw some initial conclusions. Additionally, our meta-analysis is based on observational studies only, as there are no randomized controlled trials examining this issue. Of note, all studies included, were of acceptable quality, as assessed by the Newcastle–Ottawa scale. The latter, although having its drawbacks, has been widely used by EULAR and other organizations in the development of their recommendations [59–61].

In conclusion, our SLR and meta-analysis provide evidence that, in RA, for patients with CHB treated with anti-IL-6 drugs, prophylaxis and referral to a hepatologist should be made. On the other hand, for patients with resolved hepatitis, anti-viral prophylaxis does not seem to confer additional benefit, and close monitoring is most probably a more beneficial approach. More evidence is needed to reach robust conclusions, not just for IL-6 inhibitors but also for drug categories such as the JAK-inhibitors, to enable more informed and targeted clinical decisions. Towards this end, randomized controlled trials enrolling RA patients treated with a specific class of bDMARDs who have chronic or resolved hepatitis, receiving or not receiving anti-viral prophylaxis, would be desirable. Other types of studies could also be helpful. For example, relevant data derived from big registries or multicentre (and preferably international) observational studies designed for this purpose (i.e. HBVr after exposure to specific bDMARDs) would give some answers.

## Data Availability

Data are available upon reasonable request to the corresponding author.
